# Minimally invasive versus open ileal ureter with ileocystoplasty: comparative outcomes and 5-year experience

**DOI:** 10.1186/s12894-024-01509-5

**Published:** 2024-06-10

**Authors:** Silu Chen, Xiang Wang, Zhihua Li, Xinfei Li, Guanpeng Han, Zihao Tao, Zhenyu Li, Peng Zhang, Hongjian Zhu, Kunlin Yang, Xuesong Li

**Affiliations:** 1 Department of Urology, Institute of Urology, Peking University First Hospital, Peking University, National Urological Cancer Center, Xicheng District, Beijing, China; 2 Department of Urology, Emergency General Hospital, Beijing, China; 3 Department of Urology, Beijing Jiangong Hospital, Beijing, China

**Keywords:** Ileal ureter replacement, Ileocystoplasty, Ureteral stricture with bladder contracture, Minimally invasive surgery

## Abstract

**Purpose:**

To present the experience of ileal ureter with ileocystoplasty (IUC), and compare the outcomes of IUC in minimally invasive procedures to open procedures.

**Patients and methods:**

From December 2017 to April 2023, twenty patients underwent IUC in open or minimally invasive (including laparoscopic and robotic) procedures. The baseline characteristics, perioperative data and follow-up outcomes were collected. Success was defined as relief of clinical symptoms, stable postoperative serum creatine and absence of radiographic obstruction. The perioperative and follow-up outcomes of open procedures and minimally invasive procedures were compared.

**Results:**

The etiology included pelvic irradiation (14/20), urinary tuberculosis (3/20) and surgical injury (3/20). Bilateral ureter strictures were repaired in 15 cases. The surgeries conducted consisted of open procedures in 9 patients and minimally invasive procedures in 11 patients. Compared to open procedures, minimally invasive surgeries had less median estimated blood loss (EBL) (100 ml vs. 300 min, *p* = 0.010) and shorter postoperative hospitalization (27 d vs. 13 d, *p* = 0.004). Two patients in the open group experienced grade 3 complications (sigmoid fistula and acute cholecystitis in one patient, and pulmonary embolism in another patient). Over a median follow-up period of 20.1 months, the median bladder functional capacity was 300 ml, with a 100% success rate of IUC.

**Conclusion:**

IUC is feasible in both open and minimally invasive procedures, with acceptable complications and a high success rate. Minimally invasive procedures can have less EBL and shorter postoperative hospitalization than open procedure. However, prospective studies with larger groups and longer follow-up are needed.

**Supplementary Information:**

The online version contains supplementary material available at 10.1186/s12894-024-01509-5.

## Introduction

Ureteral stricture with bladder contracture (USBC) is a relatively rare condition caused by urinary tuberculosis or radiation [1–3], posing a potential risk to renal function and diminishing overall quality of life [4]. The surgical management of extensive ureteral strictures remains a complex task, particularly when coupled with bladder contracture. Conventional reconstruction techniques such as psoas hitch and Boari flap may not be appropriate for the majority of these patients due to concerns regarding excessive tension on the anastomosis or inadequate bladder capacity [5]. Since Hubmer et al. [6] reported the first ileal ureter with ileocystoplasty (IUC) in 1988, it has become a commonly employed method for the management of USBC [7–13]. However, the intricate nature of the surgical procedure and the high incidence of complications have led to a predominance of open procedures for IUC.

Initially, we accumulated some experience in open IUC [14]. Subsequently, we successfully replicated this technique in laparoscopic and robotic ureteral surgery. In this study, we report our 5-year experience with IUC, while also comparing the perioperative outcomes of open procedures and minimally invasive procedures.

## Patients and methods

### Patients

From December 2017 to April 2023, a total of twenty patients underwent IUC, comprising seven patients who were retrospectively reviewed and thirteen patients who were prospectively enrolled. Indications for IUC included extensive or multiple ureteral strictures, either unilateral or bilateral, with bladder contracture. All surgeries were performed by an experienced surgeon (Prof. Xuesong Li). The patients’ demographics and perioperative results were collected in Reconstruction of Urinary Tract: Technology, Epidemiology and Result (RECUTTER) database (http://3dmi.com.cn). The study was approved by the institutional review board (No.: 2020-SR-283).

### Preoperative preparation

Prior to surgery, nephrostomy tubes were placed at least one month in advance, while ureteral stents were concurrently removed to facilitate adequate ureteral rest [14, 15]. . Subsequently, anterograde and retrograde urography were conducted two weeks after nephrostomy placement. Cystography was performed to evaluate the bladder volume when suspected to have bladder contracture. The patients recorded the daily volume of urine drained from the nephrostomy. Renal function was evaluated by diuretic renography using technetium-99m diethylenetriamine pentaacetic acid (^99^Tc-DTPA). Computed tomography urography (CTU) and three-dimensional reconstruction enabled better visualize the anatomy of the involved surgical area, which were employed for preoperative planning and intraoperative navigation [16, 17]. For patients with a tumor history, a comprehensive oncological assessment was essential, and tumor control was preferred for at least 3 years before contemplating ureteral reconstruction. For patients with urinary tract infection (UTI), urine samples from both the urethra and the nephrostomy were subjected to routine tests and cultures to guide antibiotic selection pre- and postoperatively. All patients underwent bowel preparation 1 day before surgery.

### Surgical technique

**Fig. 1:**
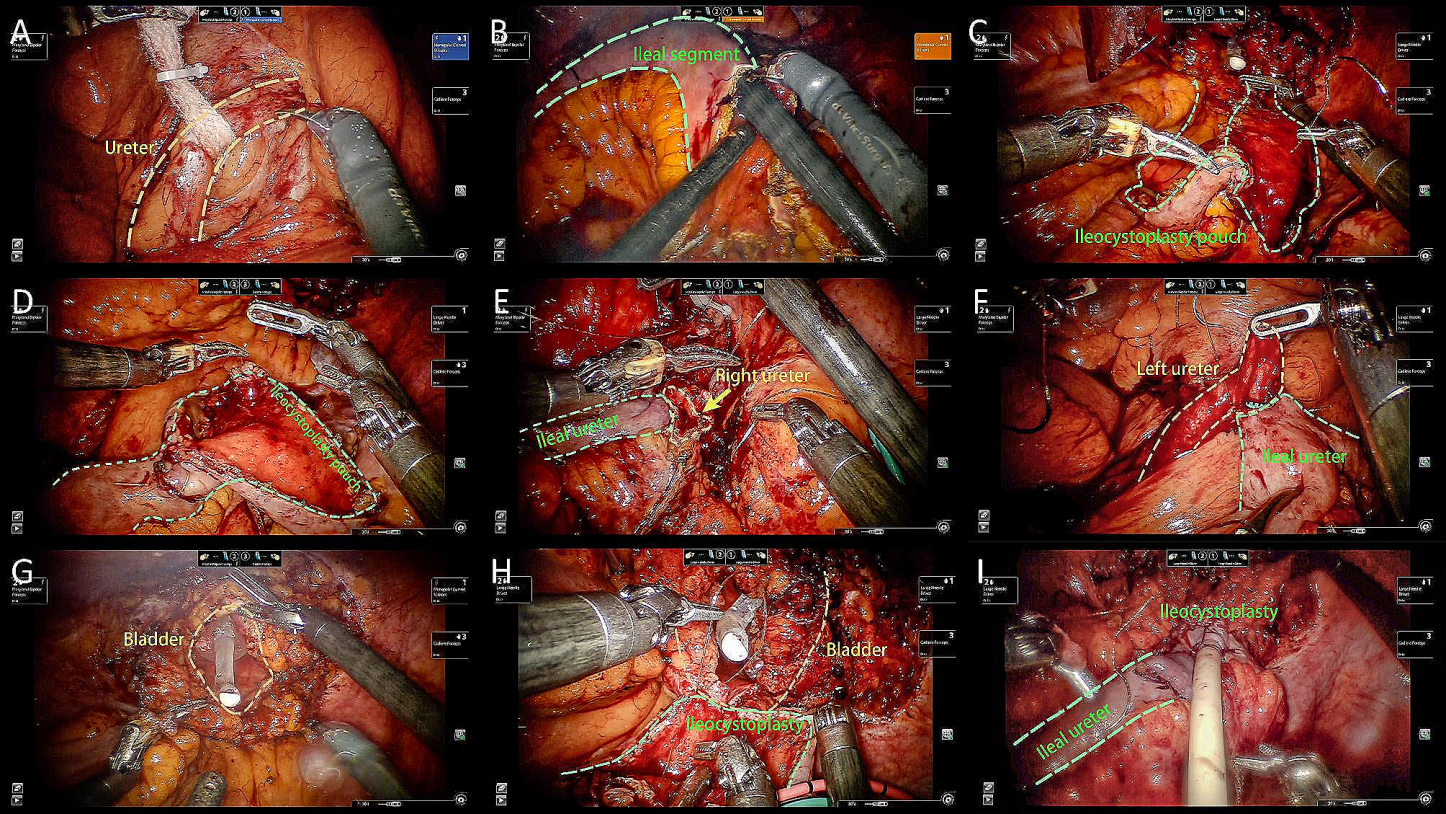
Surgical technique of ileal ureter with ileocystoplasty. **(A)** Ureteral localization. **(B)** Detubularization of the distal-most 15-cm ileum. **C-D.** Construction of a U-shaped augmentation pouch. **E.** End-to-end ileoureteral anastomosis. **F.** Contralateral end-to-side ileoureteral anastomosis for bilateral ileal ureter replacement. **G.** V-like shape cystotomy. **H-I.** Ileovesical anastomosis

**Fig. 2:**
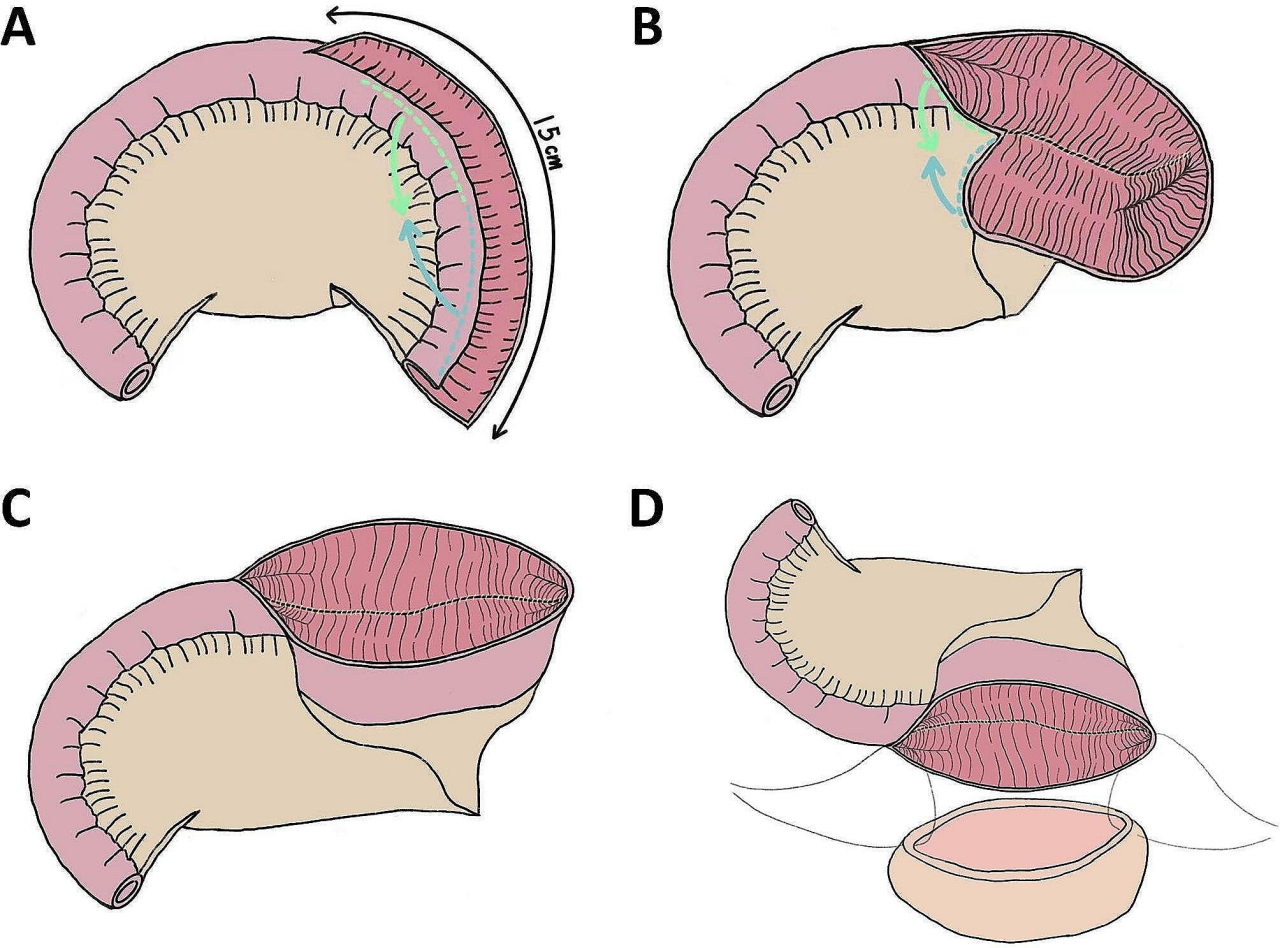
The construction of U-shaped ileocystoplasty pouch. **A.** Detubularization for 15 cm at distal ileal segment. **B-C.** Fold of the distal ileal segment with the two neighboring edges suture together **D.**Ileovesical anastomosis

As shown in **Video S1**, patients were placed in a Trendelenburg position after general anesthesia. For right side, we commonly found the ureter in the vicinity of iliac artery after mobilization of the ileocecal region. For localization of left-side ureter, the transmesocolic approach was preferred to prevent mobilization of the descending colon (Fig. 1A). The ureteral stricture was identified according to its color and texture. In robotic cases, the near-infrared fluorescence imaging technique was very useful when evaluating the blood supply of the ureter. The length of the ureter to be replaced was measured with a calibrated ureteral catheter or a premeasured 15-cm 2 − 0 silk suture approximately 20 cm proximal to the ileocecal valve. An additional 15-cm distal ileum was needed for the construction of ileocystoplasty pouch. Before isolation of the ileal segment, the ileum was marked with sutures to ensure it was isoperistaltic. Intestinal continuity was re-established in a side-to-side manner with gastrointestinal staplers [18]. After the isolated ileal segment was irrigated with diluted povidone iodine to clear the enteric contents, the distal-most 15-cm ileum was detubularized along the antimesenteric border (Figs. 1B and 2A). At the two ends and their midpoint, we often used 3 − 0 Vicryl sutures to fix the neighboring edges to avoid the crimping. The distal 15 cm ileal segment was folded, and the neighboring edges of the detubularized ileal segments were joined with 3 − 0 absorbable barbed sutures in a running suture in a U-shape (Figs. 1C-D and 2B). The distal-most transverse edge was anastomosed to the proximal detubularized edge to construct a U-shaped ileocystoplasty pouch (Fig. 2C). Thereafter, ureterotomy was performed longitudinally at 1 cm proximal to the lesion, followed by urine outflow. The proximal end of the ureter was incised 3 cm to ensure that it was consistent with the diameter of ileum. The end-to-end anastomosis of the ureter to the ileal segment was made by 4 − 0 absorbable barbed sutures in a running fashion (Fig. 1E). For bilateral ureteral reconstruction, the ileal segment was placed in a “7”, “reverse 7”, or “Y” configuration, which was determined by the location of the proximal ends of the ureteral lesion [14]. For a “7” or “reverse 7” configuration, after a 2–3 cm incision was made approximately 10 cm from the proximal end of the ileal segment, an additional end-to-side anastomosis was performed on the contralateral ureter to this incision (Fig. 1F). For a “Y” configuration, an end-to-end ileoureteral anastomosis and an ileoileal end-to-side anastomosis were needed. After mobilizing the bladder from its attachments, a transverse cystotomy was made on the posterior wall in a V-like shape (Fig. 1G). Afterward, the U-shaped ileocystoplasty pouch was sutured to the pre-dissected bladder using full-thickness sutures, and a cystotomy tube was placed (Figs. 1H-I and 2D).

### Postoperative management and follow-up

Intravenous antibiotics were administered for 5–7 days after surgery. The drainage tube was removed when the drained fluid was < 50 ml for 2 consecutive days. Bladder irrigation via the suprapubic tube was started immediately after surgery to eliminate the intestinal mucus. The nephrostomy tube was closed at 10 days. At 3 weeks, cystography was performed, and the Foley catheter was removed without concern of urine leakage. Meanwhile, the cystotomy tube was closed. The cystotomy tube and double-J stent were extracted approximately 1 month after surgery. At 3 months, upper urinary tract videourodynamics was conducted to evaluate the patency of the ileal ureter [19, 20]. In the event that videourodynamics indicated no obstruction and the patient remained asymptomatic, the nephrostomy tube was subsequently extracted.

**Fig. 3:**
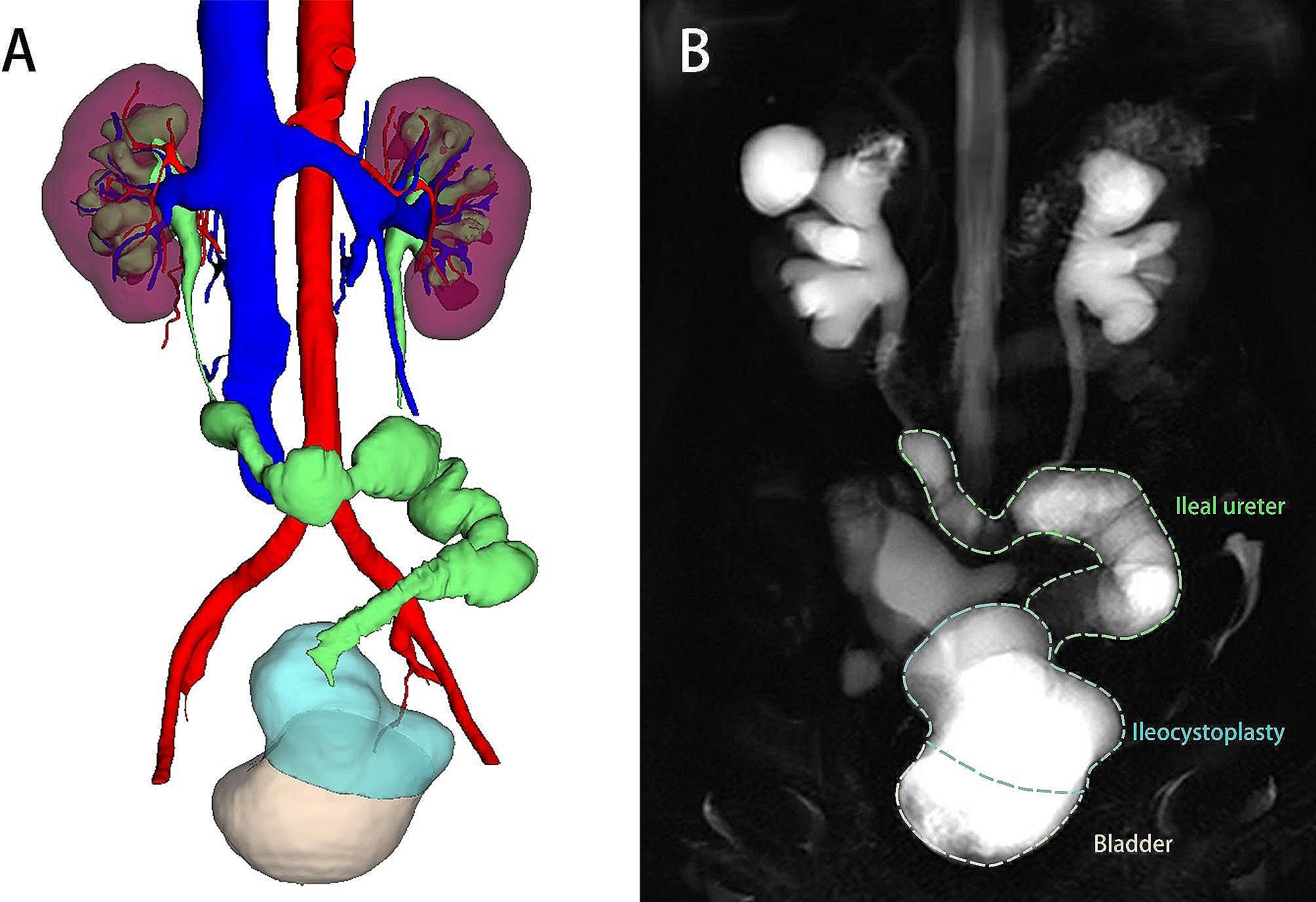
Postoperative radiographic examination during follow-up. **(A)** Postoperative three-dimensional CTU **(B)** Functional cine MRU

Blood tests (including serum creatinine, estimated glomerular filtration rate, electrolyte test, and blood gas analysis) were performed every 3 months after surgery. Sodium bicarbonate tablets was used for patients with metabolic acidosis. Functional cine magnetic resonance urography (MRU) was performed at 3 months [21, 22]. Diuretic renography and CTU were scheduled at 6 months. Thereafter, radiological examination (including CTU or cine MRU) (Fig. 3) and diuretic renography were performed every 6 months. The postoperative bladder functional capacity was measured with a measuring cup and documented in a voiding diary every month. Complications of grade 2 and above according to the Clavien-Dindo classification were recorded. The criteria for success during the follow-up period were as follows: relief of clinical symptoms, stable postoperative serum creatine level and absence of radiographic obstruction.

### Statistical analysis

Mann–Whitney 𝑈 test was used to compare continuous variables, and Fisher’s exact test was used to compare categorical variables between the open group and the minimally invasive group. *P* < 0.05 was deemed statistically significant.

## Results

The demographic characteristics are shown in Table 1. The etiology included pelvic irradiation (14/20), urinary tuberculosis (3/20) and surgical injury (3/20). Twenty USBC patients underwent IUC, including 5 unilateral cases and 15 bilateral cases. These comprised 25 middle-distal ureteral strictures, 6 full-length ureteral strictures and 4 distal ureteral strictures. The median length of ureteral strictures was 17 (range, 6–30) cm. As shown in Table 2, the surgeries conducted consisted of open procedures in 9 patients and minimally invasive procedures (including laparoscopic and robotic procedures) in 11 patients. The median operative time was 340 min, with a median estimated blood loss (EBL) of 300 ml. The median length of the ileum used for IUC was 35 cm. The median postoperative hospitalization time was 15.5 days.

**Table 1:** Demographic characteristics of USBC patients

Variables	Overall
Age (years), median (range)	49 (25–65)
Gender, n (%) Female	20 (100)
BMI (kg/m^2^), median (range)	22.3 (18.0-27.6)
Affected side, n (%)	
Left Right Bilateral	4(20) 1(5) 15(75)
Location of ureteral strictures, n (%)	
Distal	4 (11.4)
Middle-distal	25 (71.4)
Full-length	6 (17.1)
Length of ureteral strictures, median (range)	17 (6–30)
Etiology, n (%)	
Pelvic irradiation	14(70)
Urinary tuberculosis Surgical injury	3(15) 3(15)
History of nephrectomy, n (%)	2(10)

USBC = ureteral stricture with bladder contracture; BMI = body mass index

**Table 2:** Perioperative and follow-up details

Variables	Overall
Preop serum creatine (µmol/L), median (range)	92.8 (65.2–109.0)
Preop eGFR (ml/min/1.73m^2^), median (range)	60.5 (36.0-103.4)
Surgical procedure, n (%) Open Laparoscopic Robot-assisted	9 (45) 5 (25) 6 (30)
Ileal configuration of bilateral cases, n (%) Reverse “7” “7” “Y”	9 (60) 3 (20) 3 (20)
Length of the ileum used, median (range)	35 (25–45)
Operative time (min), median (range)	340 (177–520)
EBL (ml), median (range)	300 (30–800)
Immediate postop serum creatine(µmol/L), median (range)	81.2 (59.1–130.0)
Immediate postop eGFR (ml/min/1.73m^2^), median (range)	66.0 (43.0-117.5)
Postop hospitalization time (day), median (range)	15.5 (7–93)
Postoperative complications, n (%)	
Clavien-Dindo grade II Metabolic acidosis Febrile UTI Incomplete ileus Clavien-Dindo grade III Sigmoid fistula Acute cholecystitis Pulmonary embolism	10 (50) 7 (35) 2 (10) 1 (5) 1 (5) 1 (5)
Follow-up time (month), median (range)	20.1 (12.1–35.1)
Bladder functional capacity at the last follow-up (ml), median (range)	300 (150–400)
Serum creatine at the last follow-up (µmol/L), median (range)	105.0 (72.0-164.0)
eGFR at the last follow-up (ml/min/1.73m^2^), median (range)	51.4 (37.1–86.8)
Success rate, n	19/19*

eGFR = estimated glomerular filtration rate; EBL = estimated blood loss; UTI = urinary tract infection

*One patient died of recurrence of rectal cancer and was excluded from the success rate calculation

As shown in Table 3, the median EBL of the minimally invasive group was significantly smaller than that of the open group (*p* = 0.010). The median operative time was 330 min in the minimally invasive group vs. 350 min in the open group (*p* = 0.456). The minimally invasive group had a significantly shorter median postoperative hospitalization time than the open group (*p* = 0.004). Two patients in the open group encountered grade 3 complications. Specifically, one patient underwent intraoperative sigmoid injury during enterolysis, and subsequently received a one-stage suture repair. This patient developed a colonic fistula 10 days after surgery, necessitating the implementation of a transverse colostomy following the failure of 3 weeks of conservative treatment. Subsequently, this patient experienced impaired wound healing and acute cholecystitis which was managed through percutaneous cholecystostomy. This patient recovered from all complications after a cumulative postoperative hospitalization period of 93 days. Another patient developed pulmonary embolism at 9 days and subsequently recuperated following the administration of conservative support and anticoagulation therapy with enoxaparine followed by rivaroxaban.

**Table 3:** The comparison of open and minimally invasive procedures

Variables	Open procedure (*n* = 9)	Minimally invasive procedure (*n* = 11)	*P*-value
Age (years), median (range)	54 (40–59)	48 (25–65)	0.261^a^
BMI (kg/m^2^), median (range)	22.3 (19.5–27.6)	22.0 (18.0-27.1)	0.503^a^
Preop serum creatine (µmol/L), median (range)	92.5 (65.2–139.0)	100.5 (70–116)	0.710^a^
Preop eGFR (ml/min/1.73m^2^), median (range)	60.0 (36.0-91.9)	61.0 (47.6-103.4)	0.370^a^
Operative time (min), median (range)	350 (240–520)	330 (177–475)	0.456^a^
EBL (ml), median (range)	300 (200–800)	100 (30–800)	**0.010** ^**a**^
Immediate postop serum creatine(µmol/L), median (range)	87.0 (59.1–125.0)	75.3 (61.0-130.0)	0.941^a^
Immediate postop eGFR (ml/min/1.73m^2^), median (range)	71.0 (43.0-99.4)	61.9 (44.0-117.5)	1.000^a^
Postop hospitalization time (day), median (range)	27 (14–93)	13 (7–27)	**0.004** ^**a**^
Major surgical complications (CD grade ≥ 3), n (%)	2 (22.2)	0 (0)	0.189^b^
Bladder functional capacity at the last follow-up (ml), median (range)	300 (150–300)	300 (280–400)	0.206^a^
Serum creatine at the last follow-up (µmol/L), median (range)	106.5 (72.0-164.0)	95.0 (75.2–152.0)	0.657^a^
eGFR at the last follow-up (ml/min/1.73m^2^), median (range)	50.8 (38.8–86.8)	61.9 (37.1–84.7)	0.351^a^
Success rate, n	8/8*	11/11	

BMI = body mass index; eGFR = estimated glomerular filtration rate; EBL = estimated blood loss; CD = Clavien-Dindo

a. Mann-Whitney U test

b. Fisher’s exact test

*One patient died of recurrence of rectal cancer and was excluded from the success rate calculation

During a median follow-up time of 20.1 months, the median bladder functional capacity was 300 ml, and the overall success rate was 100% (19/19). One patient in the open group died of rectal cancer recurrence 22.6 months after surgery and was excluded from the success rate calculation. Postoperative CTU demonstrated that there was no evidence of obstruction in any patients. Functional cine MRU showed resolution of preoperative hydronephrosis and good peristalsis in the ileal graft. Only one patient needed clean intermittent catheterization (CIC) after surgery due to preoperative weakness of the bladder detrusor muscle caused by pelvic radiation. Another patient exhibited a urinary frequency of every half hour, with a limited bladder functional capacity of 150 ml. However, they expressed contentment with their present quality of life, as the removal of double-J stents and nephrostomy tubes was successfully accomplished without any recurrence of ureteral stricture. At the last follow-up, the median bladder functional capacity (*p* = 0.206), serum creatine level (*p* = 0.657) and eGFR (*p* = 0.351) were similar between the two groups.

## Discussion

USBC is a rare complication that may threaten renal function and disturb quality of life. IUC was initially used for the management of tuberculosis-related USBC [6]. However, as the healthcare system has progressed, the incidence of tuberculosis-related USBC has gradually declined, while USBC resulting from pelvic tumor treatments is becoming more prevalent. In the absence of surgical intervention, patients with USBC are left with the prospect of enduring permanent drainage through nephrostomy tubes or ureteral stents. Complications such as flank pain, fatigue, UTI and irritative bladder symptoms will impair patients’ quality of life if subjected to long-term drainage [3, 4].

Surgical reconstruction offers a viable treatment for protecting renal function and simultaneously improve patients’ quality of life, but surgical intervention is full of challenges. Conventional reconstruction techniques like psoas hitch or Boari flap are constrained by insufficient bladder capacity, thus rendering the utilization of bowel segments for reconstruction a potentially promising alternative. Extensive ureteral strictures can be effectively addressed through the employment of IUR [23, 24]. Additionally, ileocystoplasty can increase bladder volume and functional capacity [25]. Since Hubmer et al. [6] reported the first IUC performed on a 7-year-old girl with tuberculosis in 1988, IUC has been used for the management of USBC [7–13]. In 2016, Jeong et al. [11] conducted a study involving seven patients who underwent open IUC after radical treatment for cervical cancer. Over a mean follow-up of 38 months, it was observed that the renal function of all patients remained stable. Colon cystoplasty with IUR was described by Takeuchi et al. [26] in 2014, and this attempt made the colon an attractive option for cystoplasty when combined with IUR.

The advancement of laparoscopic and robotic technology in urology has facilitated the application of minimally invasive procedures in ureteral reconstruction [27–29]. According to our initial surgical experience of IUC [30] and previous experience of IUR [14, 18, 29, 31], care should be taken on the key points below. First, it is imperative to accurately measure the harvested ileum intraoperatively to ensure a tension-free anastomosis. An insufficient ileal segment may lead to a high-tension anastomosis, while excessive intestinal absorption caused by a lengthy ileum may result in metabolic acidosis. Second, to avoid urinary leakage and postoperative ureteral re-obstruction, a watertight and broad anastomosis with the use of 4 − 0 absorbable barbed suture in an end-to-end manner is preferred. Third, during the harvest of the ileal segment, special attention should be given to preserving the blood supply of the ileal segment from the mesentery. Finally, to prevent renal function deterioration resulting from urine reflux, it is imperative to align the direction of ileal peristalsis with the flow of urine.

Alongside surgical techniques, proper preoperative evaluation and postoperative management are crucial for a satisfactory outcome. Implementing ureteral rest, which is defined as the absence of hardware (i.e. double-J stent or percutaneous nephroureteral tube) across a ureteral stricture, prior to ureteral reconstruction may allow for stricture maturation and is associated with higher surgical success rates and lower EBL [15]. Thus, we usually placed the nephrostomy tube at least 1 month before surgery. Postoperative radiological assessment and serologic tests are regularly performed to discover potential re-obstruction and acid-base disturbance in a timely manner.

As for postoperative complications, two patients in the open group developed grade 3 complications. Metabolic acidosis occurred in half of overall patients, which may be attributed to the excessive intestinal absorption caused by the extra ileum used for ileocystoplasty. Oral sodium bicarbonate tablets were used for those patients. Febrile UTI was another disturbing complication, we used antibiotics according to routine tests and cultures of urine samples.

This study certainly had some limitations. First, approximately half of the patients were retrospectively included, and selective biases are inevitable. Second, the enrollment of patients was limited due to the low incidence of USBC, with only female patients being included in this study. Third, the follow-up time remained relatively brief. Consequently, future research necessitates prospective studies encompassing larger sample sizes and extended follow-up periods to compare the enduring outcomes of open and minimally invasive IUC.

Our research demonstrated that both open and minimally invasive IUC can be performed successfully with a good prognosis. Laparoscopy, with its enhanced visibility due to bright illumination and magnification, offers potential advantages over open procedures, particularly during ileoureteral anastomosis and ileovesical anastomosis. The traditionally large incision in open procedures is always associated with a larger scar, increased pain and slower recovery, which may contribute to reduced EBL and shorter postoperative hospitalization time. The duration of robotic docking was incorporated into the overall operative time of minimally invasive surgery. While there was no significant difference between the groups, it appeared that minimally invasive surgeries exhibited a tendency towards shorter operative time compared to open procedures (330 min vs. 350 min).

## Conclusions

The safety and feasibility of IUC was observed in both open and minimally invasive procedures, with acceptable complications and a high success rate. Furthermore, minimally invasive procedures exhibited lower EBL and shorter postoperative hospitalization durations compared to open procedures. Nevertheless, it is imperative to conduct prospective studies involving larger sample sizes and longer follow-up periods to further validate these findings.

### Electronic supplementary material

Below is the link to the electronic supplementary material.

Supplementary Material 1

## Data Availability

The data that support the findings of this study are available from the corresponding author upon reasonable request.

## Abbreviations

BMI: Body mass index
CTU: Computed tomography urography
EBL: Estimated blood loss
eGFR: Estimated glomerular filtration rate
IUR: Ileal ureter replacement
IUC: Ileal ureter with ileocystoplasty
MRU: Magnetic resonance urography
USBC: Ureteral stricture with bladder contracture
UTI: Urinary tract infection
